# A rare case of pericarditis and pleural empyema secondary to transdiaphragmatic extension of pyogenic liver abscess

**DOI:** 10.1186/s12879-018-2953-8

**Published:** 2018-01-15

**Authors:** Eunae Cho, Sang Woo Park, Chung Hwan Jun, Sang Soo Shin, Eun Kyu Park, Kyo Seon Lee, Seon Young Park, Chang Hwan Park, Hyun Soo Kim, Sung Kyu Choi, Jong Sun Rew

**Affiliations:** 1 Department of Internal Medicine, Chonnam National University Hospital, Gwangju, South Korea; 2 Department of Radiology, Chonnam National University Hospital, Gwangju, South Korea; 3 Department of Surgery, Chonnam National University Medical School, Gwangju, South Korea; 4 Department of Thoracic and Cardiovascular Surgery, Chonnam National University Hospital, Gwangju, South Korea

**Keywords:** Liver abscess, Pericarditis, Empyema, *Klebsiella pneumoniae*

## Abstract

**Background:**

Transdiaphragmatic extension of pyogenic liver abscess is the rarest cause of pericarditis and pleural empyema. It is a rapidly progressive and highly lethal infection with mortality rates reaching 100% if left untreated. However, the transmission route, treatment methods and prognosis have not been well studied.

**Case presentation:**

A 65-year-old male patient presented with a fever, dyspnea, and right upper quadrant abdominal pain. Computed tomography of the chest and abdomen showed huge liver abscess without full liquefaction in the left lobe, large amount of left pleural effusion, and mild pericardial effusion, and the patient was treated with parenteral antibiotics and pigtail insertion at the left pleura. However, four days later, cardiac tamponade was developed and surgical drainage of the abscess and pericardium was performed. *Klebsiella pneumonia* was isolated from pleural empyema. Twenty-five days after surgery, the patient was discharged without any complications.

**Conclusions:**

Herein, we report a rare case of pleural empyema and pericarditis in that resulted from the extension of huge pyogenic liver abscess. Early surgical treatment may have prevented progression of the pericarditis to the more dismal purulent pericarditis. We also review pertinent English literature on pericarditis as a complication of PLA.

## Background

Pyogenic liver abscess (PLA) is a life-threatening infectious condition, with an estimated incidence of 2.3 cases per 100,000 persons in the United States [1] and 17.6 cases per 100,000 persons in Taiwan [2]. Despite aggressive treatment, the mortality rate ranges from 6% to 14% [3]. *Klebsiella pneumoniae* is an important cause of PLA in Asia, and is often associated with invasive liver abscess syndrome, which accompanies metastatic infection at other sites [4, 5]. However, pericarditis by direct PLA invasion has rarely been reported [6]. Herein, we report one case of a transdiaphragmatic extension of a *Klebsiella pneumoniae* liver abscess, which caused pleural empyema and pericarditis. We also review pertinent English literature on pericarditis as a complication of PLA.

## Case presentation

**Fig. 1:**
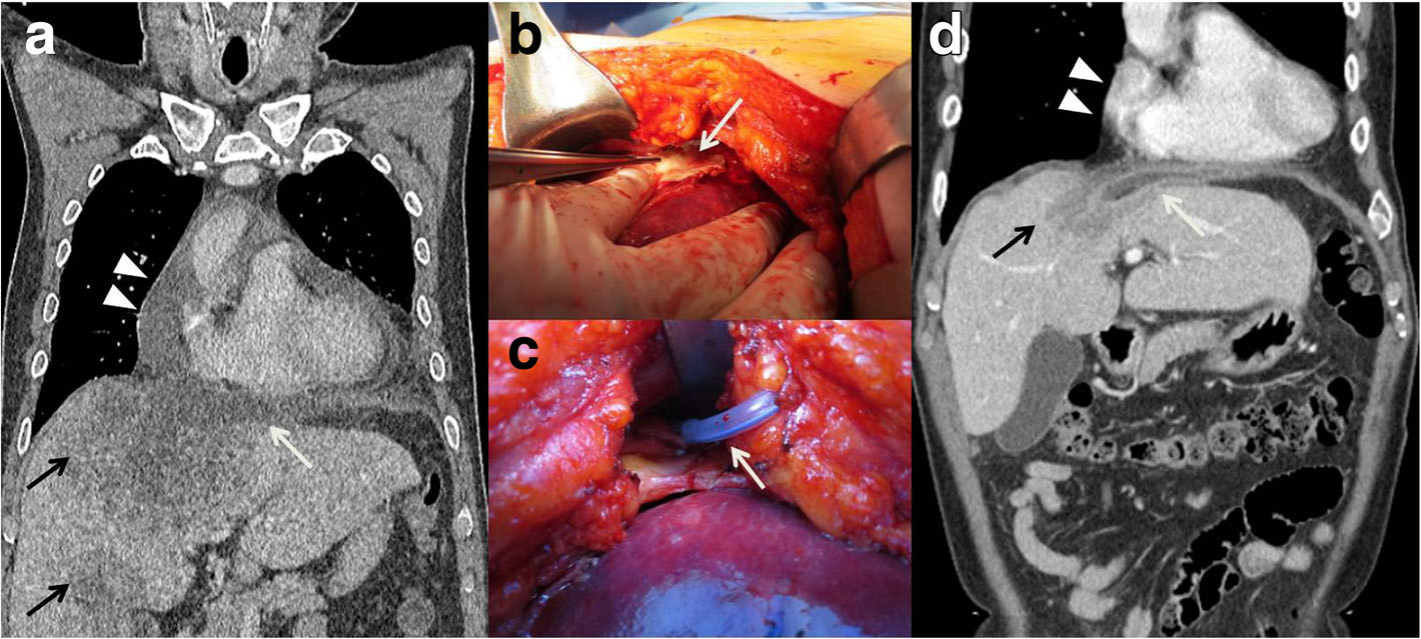
**a**. Computed tomography (CT) of chest demonstrated 10 × 6 × 8 cm (segment IV) and 4 × 4 × 4 cm (segment V) sized liver abscesses (black arrows), subphrenic fluid collection (white arrow), and moderate amount of pericaridal effusion (arrowheads). **b**. Intraoperative findings showed inflamed and necrotic diaphragm (white arrow) abutting liver abscess. **c**. After pericardial window was created, a pericardial drainage tube was inserted (white arrow). **d**. Follow-up abdomen CT after 20 days showed decreased size of liver abscess (black arrow) and subphremic fluid collection (white arrow), and complete resolution of pericardial effusion (arrowheads)

A 65-year-old man was transferred to our hospital due to dyspnea. Five days prior to his admission, he had presented to another hospital with right upper quadrant abdominal pain accompanied by fever, and a diagnosis of liver abscess was made. He denied any ophthalmological symptoms such as blurred vision or painful eye swelling. The patient was treated with parenteral ceftriaxone and metronidazole, but his condition deteriorated. On admission, his vital signs were as follows: pulse, 122 beats/min; blood pressure, 100/60 mmHg; respiratory rate, 24 breaths/min; and temperature, 38.0 °C. On physical examination, decreased breath sounds in the left lower lung fields and tenderness over the right upper abdomen were noted. Laboratory blood tests revealed leukocytosis, elevated liver enzymes (AST 42 U/L, ALT 74 U/L, ALP 162 U/L, and r-GTP 114 U/L) and a C-reactive protein level of 18.2 mg/dL. A computed tomography (CT) scan of the abdomen revealed one 10x6x8 cm and one 4x4x4 cm liver abscess, without full liquefaction, in the hepatic dome and anterior medial segment, respectively, and a small amount of ascites under the diaphragm. A chest CT revealed a small right pleural effusion, a large left pleural effusion and mild pericardial effusion. A diagnostic thoracentesis was performed. The aspirated pleural fluid was grossly pus (pH, 7.009; LDH, 7974 U/L; and protein, 3.3 g/dL). A pigtail catheter was inserted into the left pleural cavity to drain the empyema. The parenteral ceftriaxone and metronidazole treatment was changed to piperacillin/tazobactam. However, the patient remained febrile and tachypneic and, after four days, he progressed to septic shock. Physical examination revealed jugular venous distension and attenuated heart sounds. The electrocardiogram (ECG) showed multiple premature ventricular complexes. Chest CT revealed an increased volume of the pericardial effusion, with pericardial enhancement (Fig. 1a). Emergent bedside echocardiography showed a moderate amount of pericardial effusion, with some refractile densities in the fluid, suggestive of possible purulent pericarditis. A decision to proceed with surgical drainage of the liver abscess and surgical pericardiostomy was made on post-admission day 5. The abdominal cavity and liver were examined first. The huge abscess in the hepatic dome was adherent to the diaphragm. Moreover, the diaphragm and chest wall were inflamed and necrotic, with loculated pus between the diaphragm and the liver abscess (Fig. 1b). The diaphragm was released from the abscess and, after unroofing of the abscess, a drain was inserted into the liver abscess. Subsequently, after partial resection of the xiphoid process, a pericardial window was created (Fig. 1c), and the pericardial fluid, which was serosanguineous, was aspirated. In addition, inflammation of the pericardium was noted and a drain was inserted into the pericardium. After surgical drainage was performed, the patient’s condition improved and the ECG normalized. The pleural fluid culture was positive for *Klebsiella pneumoniae*; however, blood, pericardial fluid and liver abscess cultures were negative. Based on antibiotic susceptibility test, piperacillin/tazobactam was changed to ciprofloxacin. Twenty-five days after surgery, the patient was discharged without any complications. Follow-up abdominal ultrasonography performed at 20 days post-discharge showed a decrease in the size of the liver abscess and subphrenic fluid collection, and complete resolution of the pericardial effusion (Fig. 1d).

## Discussion and conclusions

Acute pericarditis is a rare but potentially lethal complication of a liver abscess, with an incidence rate of < 2% in patients with an amebic liver abscess [4]. PLA resulting in pericarditis is a rarer occurrence. In our report, we describe a very rare case of a huge liver abscess in the left lobe of the liver that extended transdiaphragmatically, causing a pleural empyema and pericarditis. Although the pleural empyema was successfully managed with insertion of a percutaneous pigtail catheter, the liver abscess could not be initially drained percutaneously as it was not fully liquefied. Despite adequate antibiotic treatment, which was later confirmed to be appropriate for the causative pathogen *Klebsiella pneumoniae,* pericarditis developed. Inflammation of the diaphragm and pericardium was confirmed during the surgery. Early surgical treatment may have prevented progression of the pericarditis to the more dismal purulent pericarditis.

A PUBMED search of the English literature identified only a few cases of pericarditis as a complication of PLA [6–17], which we have summarized in Table 1. The median age of these patients was 49 years (range, 20 ~ 73 years), with 8 of the 13 patients (61.5%) being male. The liver abscess was located in the left lobe of the liver in 10 cases (76.9%) [6, 7, 9, 11–17] and in the right lobe (segment V/VIII) in one case (7.7%) [8], with involvement of both right and left lobes in one case (7.7%) [10]. In our case, the abscess was located in the left lobe. The transmission route of the PLA to the pericarditis was transdiaphragmatic in most of the previously reported cases [6, 10–15, 17], including our case.

**Table 1:** Review of thirteen cases of pericarditis complicated by pyogenic liver abscess in English literature

Year	Age /sex	Comorbidites	Cardiac complications	Location of liver abscess	Pathogen	Positive culture site	Transmission route	Treatment other than antibiotics	Clinical outcomes
2015 [6]	44/M	Homeless, Cholecystostomy state	Empyema, Purulent pericarditis, cardiac tamponade	Lt. lobe	*K. pneumoniae*, *C. glabrata*	Pericardial fluid	Trans-diaphragmatic	Surgical pericardial drainage, hepatic abscess resection, cholecystostomy tube removal	Recovery
2015 [7]	23/M	No	Myopericarditis	Lt. lobe	*F. nucleatum*	Blood, hepatic abscess	Reaction to liver abscess	Percutaneous abscess drainage	Recovery
2014 [8]	49/F	Rectal cancer with hepatic metastasis	Purulent pericarditis, cardiac tamponade	Segment V/VIII	*E .faecalis, E. coli*	Pericardial fluid, surgical site abscess, laparotomy wound	N/A	Pericardiocentesis, surgical abscess drainage	Recovery
2012 [9]	20/M	Subtotal esophagectomy	Purulent pericarditis, cardiac tamponade	Lt. Lobe	*Actinomyces* spp., *Fusobacterium* spp., *Peptostreptococcus* spp.	Pericardial fluid, hepatic abscess	N/A	Pericardiocentesis, percutaneous abscess drainage	Recovery
2012 [10]	60/M	P/H of Pul. TB & alcoholic pancreatitis	Purulent pericarditis, cardiac tamponade	Rt. & Lt. Lobes	*Actinomyces* spp.^a^, *K.oxytoca, C. koseri*	Pericardial fluid	Trans-diaphragmatic	Pericardiocentesis, video-assisted thoracic surgery	Recovery
2010 [11]	62/F	HTN, IHD	Purulent pericarditis, cardiac tamponade	Lt. Lobe	*E. coli*	Pericardial fluid	Trans-diaphragmatic	Subxiphoid pericardial window and drainage	Death
2008 [12]	73/M	A.fib, OA	Purulent pericarditis, cardiac tamponade	Lt. Lobe	*K. pneumoniae*	Pericardial fluid, hepatic abscess	Trans-diaphragmatic	Pericardiocentesis, percutaneous abscess drainage	Death
2007 [13]	73/F	No	Purulent pericarditis, cardiac tamponade	Lt. Lobe	*E. coli*	Blood, pericardial fluid, hepatic abscess	Trans-diaphragmatic	Pericardiocentesis, percutaneous abscess drainage	Recovery
2007 [14]	73/M	OA on steroid	Purulent pericarditis, cardiac tamponade	Lat. segment of Lt. lobe	*K. pneumoniae*	Pericardial fluid, hepatic abscess	Trans-diaphragmatic	Pericardiocentesis, percutaneous abscess drainage	Death
	65/M	DM, CKD	Purulent pericarditis, cardiac tamponade	Lat. segment of Lt. lobe	*K. pneumoniae*	Pericardial fluid, hepatic abscess	Trans-diaphragmatic	Pericardiocentesis, percutaneous abscess drainage	Recovery
2006 [15]	N/A	N/A	Purulent pericarditis, cardiac tamponade	Lt. lobe	*Proteus* spp., *Enterococcus* spp.	Pericardial fluid	Trans-diaphragmatic	Pericardiocentesis, thoracotomy, pericardiectomy	N/A
2002 [16]	48/F	No	Purulent pericarditis, cardiac tamponade	Lt. lobe	*M. morganii*	Blood, pericardial fluid, hepatic abscess	N/A	Pericardiocentesis, thoracotomy, pericardiectomy	Recovery
1996 [17]	32/M	No	Purulent pericarditis, cardiac tamponade	Lt. lobe	*E. coli*	Blood, pericardial fluid	Trans-diaphragmatic	Pericardiotomy, hepatic abscess drainage	Recovery

Lt., left; Rt., right; Lat., lateral; N/A, not available; P/H, past history; Pul. TB, pulmonary tuberculosis; HTN, hypertension; IHD, ischemic heart disease; A.fib, atrial fibrillation; OA, osteoarthritis; DM, diabetes mellitus; CKD, chronic kidney disease; *K. pneumoniae, Klebsella pneumoniae; C. glabrata, Candida glabrata; F. nucleatum, Fusobacterium nucleatum; E. faecalis, Enterococcus faecalis; E. coli, Escherichia coli; K. oxytoca, Klebsiella oxytoca; C. koseri, Citrobacter koseri; M. morganii, Morganella morganii*

^a^Hepatic Actinomycosis was diagnosed based on the pathological examination of liver abscess

In previously reported cases of PLA with pericarditis, patients complained of various symptoms including a fever, abdominal pain, dyspnea, and chest pain [6–17]. Tachycardia and septic shock were usually present. Distention of the jugular veins or an elevated jugular pressure are the most important finding suggestive of a pericardial involvement, due to the development of septic shock, which usually causes the central venous pressure to fall and the jugular veins to collapse. This finding was positive in twelve patients (92.3%) [6, 8–17], and in our case as well. All of these patients developed a cardiac tamponade that can be fatal if left untreated. Therefore, early identification is crucial. However, ECG findings in these cases can vary, including ST segment elevations [6, 7, 17], low voltage in limb leads [12] and ventricular tachycardia [14], or the ECG can be normal [15]. In our case, the ECG showed multiple premature ventricular complexes. An enlarged cardiac silhouette is frequently observed on chest X-rays [6, 11, 14, 16, 17], with or without pleural effusion. On CT imaging of the abdomen or chest, liver abscesses with pericardial effusion are a frequent finding [6, 8–10, 12–15]. Therefore, if patients with liver abscess complain of dyspnea or chest pain and distended jugular veins are noted, a thorough evaluation, including chest CT or transthoracic echocardiography, for pericardial involvement is needed.

In terms of causative pathogen for PLA associated with pericarditis, *K. pneumoniae* was identified in four cases (30.7%) [6, 12, 14], *E.coli* in four cases (30.7%) [8, 11] and *Actinomyces spp*. [9, 10] in two cases (15.4%). Other responsible pathogens previously reported include *M. morganii* [16], *K. oxytoca* [10], *C. koseri* [10]*, Candida* [6], *Enterococcus* [8, 15], *Fusobacterium* [7, 9], *Peptostreptococcus* [9], and *Proteus* [15]. Although various pathogens have been described for the cause of PLA, *K. pneumoniae* is the most common responsible pathogen in Asia [5]. Jun et al. have reported *K. pneumoniae* as the most commonly isolated organism in the abscess (49.8%), followed by Streptococcus species (3.8%) and *E. coli* (3.3%). *K. pneumoniae* was also the most commonly isolated organism in blood (70.2%), followed by *E. coli* (10.7%) [18]. However, the most common pathogen associated with bacterial pericarditis is not *K. pneumonia.* In one study involving 933 acute pericarditis patients, 32 patients had bacterial origin, and staphylococci (21.9%) and streptococci (15.6%) were the most common organisms. *K. pneumoniae* was responsible for only 3.1% of cases [19]. It is very interesting that *K. pneumoniae* was the most common pathogen for PLA associated with pericarditis including our case.

It has been reported that K1 strains of *K. pneumoniae* are associated with PLA complicated by metastatic endophthalmitis or CNS infections [20], and Yu et al. have reported a case of bacterial pericarditis by genotype K1 [21]. Endophthalmitis is the most common metastatic complication of PLA, and the incidence is reported to be 0.84–1.92%. Symptoms of endophthalmitis are visual impairment leading to blindness, or painful eye swelling. Suggested risk factors of endophthalmitis include diabetes mellitus, K1 strains of *K. pneumoniae,* abscess in the right superior segment, and other systemic infection. Intravitreal antibiotics and early vitrectomy are the mainstay of the treatment, but the prognosis is poor [4].

The K1 strain has been reported to be highly virulent because of hypermucoviscosity, which is associated with high serum resistance, high-level resistance to phagocytosis, and resistance to complement deposition [20, 21]. Although the genotypic analysis has not been performed in the reported cases, it is possible that highly virulent strains of *K. pneumoniae* were associated with PLA complicated by pericarditis. Thus, further studies are needed to identify virulent strains that are responsible for PLA associated with pericarditis.

Among the 12 cases reporting purulent pericarditis as a complication of PLA, pericardial fluid culture was positive in all patients [6, 8, 9, 11–17]. In contrast, the pericardial fluid in our case did not reveal any microorganism. This is probably because early detection and surgical treatment of pericarditis prevented progression to purulent pericarditis. Although PLA was the primary infection focus, only eight cases (61.5%) reported positive cultures of hepatic abscess [7–9, 12–14, 16]. Rates of positive blood cultures were even lower, with only four (30.8%) reported cases [7, 13, 16, 17]. Both hepatic abscess and blood culture results were negative in our case. The negative culture from hepatic abscess may be due to the delay in obtaining the culture (nine days after initial antibiotic treatment) as the initial percutaneous aspiration was not possible. However, the negative blood culture is noteworthy in light of such an overwhelming infection, with positive blood cultures typically reported in 50% of cases of PLA [22].

The following treatments for PLA-associated pericarditis have been reported: pericardiocentesis in six cases (46.1%) [8, 9, 12–14]; surgical pericardial drainage or pericardiectomy in six cases (46.1%) [6, 10, 11, 15–17], combined with percutaneous abscess drainage in seven cases (53.8%) [7, 9, 12–14, 17]; and surgical abscess resection in two cases (15.4%) [6, 8]. All patients received antibiotic treatments. Three patients (23%) died among the previously reported cases, indicative of the higher mortality rate for PLA-associated pericarditis than the reported mortality rate of 5–10% for PLA alone [23].

In conclusion, pericarditis is a rare but fatal complication of left lobe liver abscess. Pericarditis should be highly suspected in patients with cardiac symptoms, jugular vein engorgements and enlarged cardiac silhouette on chest X-ray. Early diagnostic evaluation, using chest CT or transthoracic echocardiography, and immediate treatment, including pericardiocentesis or pericardiotomy and abscess drainage, combined with intravenous antibiotics can lower the risk of patient death.

## Abbreviations

ALP: Alkaline phosphatase
ALT: Alanine Aminotransferase
AST: Aspartate Aminotransferase
*C. glabrata*: *Candida glabrata*
*C. koseri*: *Citrobacter koseri*
CT: Computed tomography
*E. coli*: *Escherichia coli*
*E. faecalis*: *Enterococcus faecalis*
ECG: Electrocardiogram
*F. nucleatum*: *Fusobacterium nucleatum*
*K. oxytoca*: *Klebsiella oxytoca*
*K. pneumonia*: *Klebsella pneumoniae*
LDH: Lactate dehydrogenase
*M. morganii*: *Morganella morganii*
PLA: Pyogenic liver abscess
r-GTP: Gamma Glutamyl Transpeptidase
