# Correction to: A rare case of pericarditis and pleural empyema secondary to transdiaphragmatic extension of pyogenic liver abscess

**DOI:** 10.1186/s12879-020-05232-y

**Published:** 2020-07-08

**Authors:** Eunae Cho, Sang Woo Park, Chung Hwan Jun, Sang Soo Shin, Eun Kyu Park, Kyo Seon Lee, Seon Young Park, Chang Hwan Park, Hyun Soo Kim, Sung Kyu Choi, Jong Sun Rew

**Affiliations:** 1 Department of Internal Medicine, Chonnam National University Hospital, Gwangju, South Korea; 2 Department of Radiology, Chonnam National University Hospital, Gwangju, South Korea; 3 Department of Surgery, Chonnam National University Medical School, Gwangju, South Korea; 4 Department of Thoracic and Cardiovascular Surgery, Chonnam National University Hospital, Gwangju, South Korea

**Correction to: BMC Infect Dis (2018) 18:40**

**https://doi.org/10.1186/s12879-018-2953-8**

After the publication of the original article [1], an error was identified in Reference 4.

The reference currently reads:

Park IH, Jun CH, Wi JW, Park SY, Lee WS, Jung SI, Park CH, Joo YE, Kim HS, Choi SK, et al. Prevalence of and risk factors for endogenous endophthalmitis in patients with pyogenic liver abscesses. Korean J Intern Med. 2015;30(4):453–9.

The reference should read:

Chest. Thoracic Complications of Amebic Abscess of the Liver: Report of 501 Cases, 1981 Jun;79(6):672–7, doi: 10.1378/chest.79.6.672.
